# Editorial: Inflammation and organic damage in COVID-19: what have we learned 2 years into the pandemic?

**DOI:** 10.3389/fmed.2023.1238804

**Published:** 2023-07-11

**Authors:** Luis García de Guadiana-Romualdo, David Andaluz Ojeda

**Affiliations:** ^1^Clinical Analysis Department, Santa Lucía University General Hospital, Cartagena, Spain; ^2^Critical Care Area, HM University Sanchinarro Hospital, HM Hospitales, Madrid, Spain

**Keywords:** long COVID, COVID-19, endothelial dysfunction, endotheliitis, inflammation, COVID-19 biomarkers

Acute coronavirus disease 2019 (COVID-19) presents a wide spectrum of clinical manifestations, from asymptomatic infection to severe pneumonia or multisystemic failure. In addition, nearly 3 years after the pandemic, now it is known that there are persistent forms of COVID-19, known as long-COVID, with long-term effects in different organs and systems. These complications related to SARS-CoV-2 infection, which significantly affect the quality of life of many convalescent patients, are not restricted to severe presentations of COVID-19; hence, many patients with persistent symptoms have never been hospitalized. The mechanisms explaining long COVID are not yet well delimited. Recent findings related to immunity alterations together with inflammation and endothelial damage induced by the virus, along with certain predisposing factors, would favor the development of these complications.

In this regard, the implication of the ABO blood group in the COVID-19 disease was formulated early at the beginning of the pandemic, and it has now been established that the A blood group is associated with more susceptibility and severe symptoms of COVID-19, while the O blood group shows protection against viral infection (1). Tamayo-Velasco et al. detail in a complete review how the presence of anti-antigen A and B antibodies in group 0 patients confers a protective effect against protein S of the virus, which could open new avenues for prognostic and therapeutic stratification. The presence of a high viral load in some individuals determines the status of persistent viraemia, which has also been shown to be an independent factor associated with bad prognosis in COVID-19 (2). In this sense, in a short prospective study, Roy-Vallejo et al. report how the presence of detectable viremia in some patients is associated with a greater inflammatory response characterized by an increase in IL-6 levels and poor evolution. Following this line, in an interesting prospective study, Melhorn et al. prove persistence of inflammatory and vascular mediators 5 months after hospitalization in a cohort of COVID-19 patients compare with healthy and septic controls. In fact, IL-6 again, along with TNF, SAA, CRP, Tie2, Flt-1, and PIGF, was significantly increased in the post-COVID group.

The post-acute sequelae of COVID-19 (PACS) represent a heterogeneous group of symptoms characterized by cardiovascular, general, respiratory, and neuropsychiatric sequelae. PACS can be classified into two categories: PACS cardiovascular disease, characterized by a group of cardiovascular conditions that develop during the chronic phase of the disease, and PACS cardiovascular syndrome (PACS-CVS), which lacks clear evidence of cardiovascular disease (3). In this Research Topic, Aparisi et al. provide insights into the role of the cardiopulmonary exercise test (CPET) in evaluating PACS-CVS. However, it is important to note that there is a lack of evidence-based recommendations for managing this elusive condition. Nonetheless, CPET should be implemented due to its ability to assess the pathophysiology of exercise limitation.

In about 25% of patients with severe COVID-19 disease (WHO Severity Grade 3 and 4), a restrictive ventilatory defect has been revealed. This and other facts justify that a significant percentage of COVID-19 patients present respiratory failure not only during the acute illness of the disease but also chronically, months after overcoming it. SARS coronavirus induces the upregulation of type I collagen (4). At 1 year after ICU admission in a cohort of 105 critically ill patients from several Spanish hospitals, in an interesting prospective multicenter study, González et al. have found that 32.2% of these patients persisted with respiratory alterations, 10% still had moderate/severe lung diffusion (DLCO) involvement (<60%), and 53.7% had a fibrotic pattern on CT. Moreover, patients had a mean (SD) number of symptoms of 5.7 ± 4.6, and 61.3% met the criteria for post-COVID syndrome at 1 year. Thus, there is a compelling clinical need to identify circulating fibrosis markers in COVID-19 leading to pulmonary pro-fibrotic responses that can identify candidate patients suffering from long-term COVID with respiratory alterations. In this regard, Brusa et al. report another circulating biomarker, known as the Targeting Matrix Metalloproteases Pathway-1 (TIMP-1), which has been associated with disease severity and the systemic inflammatory index, suggesting a promising non-invasive prognostic biomarker for structural respiratory damage in COVID-19 patients.

Beyond the local and systemic inflammatory response, endothelial dysfunction (ED) or endotheliitis has been demonstrated to play a critical role in COVID-19 acute organ disfunction and may also be related to long-term systemic symptoms. ED favors both inflammatory activation and local coagulation, leading to hypercoagulability states (HS), microthrombosis, and hypoperfusion, more markedly in microcirculation (5). Due to this, cardiovascular pathologies such as myocardial damage and thromboembolic events (TE) have been frequently related to COVID-19 (6). In a comprehensive review, Izquierdo-Marquisá et al. detail how myocardial injury is present in around one-third of hospitalized COVID-19 patients, and this condition is associated with worse in-hospital outcomes, with over 50% mortality. Myocardial injury-related mechanisms are varied (myocarditis related to viral infection, ED, or HS), and quick identification is key to being able to treat it early. Beyond the classic diagnostic tests of myocardial injury (electrocardiogram and echocardiogram) and cardiac biomarkers (such as troponin and natriuretic peptides), the identification of new affordable and bedside biomarkers seems essential to identify this potentially fatal situation. Recent studies have evaluated the role of MR-proadrenomedullin (MR-proADM), a novel marker of ED in sepsis and pneumonia (7, 8). It is a pro-hormone with vasodilator properties synthesized by endothelial cells. High levels of MR-proADM achieved an excellent accuracy to predict mortality and poor outcome in patients with COVID-19 (9). In this sense, Spoto et al. demonstrate how this molecule complements troponin, a canonical biomarker of myocardial damage, improving its prognosis accuracy and risk stratification in a cohort of COVID-19 patients with myocardial injury. Despite the rationale that early antiplatelet therapy would lower the risk of cardiovascular events on the basis of their antithrombotic and anti-inflammatory properties, the effectiveness of this approach remains controversial (10). In this regard, Zong et al. perform a systematic review and meta-analysis, including early observational studies and recent randomized controlled trials (RCTs) assessing the effect of antiplatelet therapy in adult patients with COVID-19. Based on 23 observational studies, including 87,824 COVID-19 patients, antiplatelet treatment has been found to favor a lower risk of mortality (odds ratio: 0.72, 95% confidence interval: 0.61–0.85; *p*-value < 0.01). However, the narrative synthesis of RCTs showed conflicting evidence, which did not support adding antiplatelet therapy to the standard care. This discrepancy seems to suggest that there are subgroups of COVID-19 patients who could benefit from this therapy, while in others such a benefit would not exist. It is necessary to carry out new and larger RCTs that evaluate antiplatelets from an individualized or personalized point of view based on the endotype of the candidate patient. In this respect, biomarkers of TE can be useful. D-dimer has shown to be a robust predictor associated with bad outcomes in COVID-19 (11). Interestingly, in a multicenter study, Ronderos Botero et al. demonstrate how the D-dimer prognostic value has also not varied in successive pandemic waves. Thus, TE biomarkers can be useful, not only at the prognostic level but also to individualize treatments.

The association of COVID-19 with prevalent gastrointestinal distress, characterized by the fecal shedding of SARS CoV 2 RNA or persistent antigen presence in the gut, has been scarcely evaluated (12). In this Research Topic, Moon present a review addressing gastrointestinal symptoms and describing data on the gut–lung axis, viral transmission to the gut, and its influence on gut mucosa and the microbial community.

We hope that this Research Topic provides original information to the scientific community on the “hot” topic of long COVID and the medium- and long-term effects of SARS-CoV-2 infection.

## Author contributions

DA drafted the manuscript. LG and DA critically revised the manuscript for important intellectual content. All authors contributed to the article and approved the submitted version.
